# Two-center randomized controlled trial comparing oral chloral hydrate and intranasal combination of dexmedetomidine and ketamine for procedural sedation in children: study protocol

**DOI:** 10.1186/s13063-022-07033-x

**Published:** 2023-01-03

**Authors:** Young-Eun Jang, Eun-Young Joo, Ji-Hyun Lee, Eun-Hee Kim, Pyoyoon Kang, Jung-Bin Park, Hee-Soo Kim, Jin-Tae Kim

**Affiliations:** 1 Department of Anesthesiology and Pain Medicine, Seoul National University Hospital, Seoul National University College of Medicine, #101 Daehak-ro, Jongno-gu, Seoul, 03080 Republic of Korea; 2 Department of Anesthesiology and Pain Medicine, Asan Medical Center, University of Ulsan College of Medicine, Seoul, Republic of Korea

**Keywords:** Dexmedetomidine, Chloral hydrate, Intranasal, Ketamine, Pediatric sedation

## Abstract

**Background:**

Oral chloral hydrate is widely used in pediatric sedation. Intranasal dexmedetomidine has been increasingly used for pediatric sedation; however, its improvement is warranted. The combination of dexmedetomidine with ketamine can improve onset and hemodynamic stability while maintaining sedative efficacy. This study aims to determine the efficacy and safety of intranasal combination of dexmedetomidine and ketamine compared to oral chloral hydrate.

**Methods:**

This is a prospective, parallel-arm, single-blinded, two-center, superiority randomized controlled trial with 1:1 allocation, designed to compare the effects of intranasal combination of dexmedetomidine and ketamine with those of oral chloral hydrate. We shall enroll 136 patients aged < 7 years old in this study. Prior to the procedure, we shall randomize each patient into the control group (oral chloral hydrate 50 mg/kg) or study group (intranasal dexmedetomidine 2 μg/kg and ketamine 3 mg/kg). The primary outcome will be the rate of achieving an adequate sedation level (6-point Pediatric Sedation State Scale 1, 2, or 3) within 15 min. In addition, we shall measure the sedation time, sedation failure rate, completion of procedure, adverse events, patient acceptance, and physician satisfaction.

**Discussion:**

This study will provide evidence of the efficacy and safety of the intranasal combination of dexmedetomidine and ketamine in comparison with oral chloral hydrate.

**Trial registration:**

ClinicalTrials.gov, NCT04820205. Registered on 19th March 2021

## Administrative information

**Table:** 

Title {1}	Two-center randomized controlled trial comparing oral chloral hydrate and intranasal combination of dexmedetomidine and ketamine for procedural sedation in children: study protocol
Trial registration {2a and 2b}.	ClinicalTrials.gov, NCT04820205. Registered on 19 March 2021 https://clinicaltrials.gov/ct2/show/NCT04820205
Protocol version {3}	Protocol ver 1.3 (August 11, 2021)
Funding {4}	This research was supported by a grant of Patient-Centered Clinical Research Coordinating Center (PACEN) funded by the Ministry of Health & Welfare, Republic of Korea (grant number : HC20C0060).
Author details {5a}	Young-Eun Jang^1^, Eun-Young Joo^2^, Ji-Hyun Lee^1^, Eun-Hee Kim^1^, Pyoyoon Kang^1^, Jung-Bin Park^1^, Hee-Soo Kim^1^ and Jin-Tae Kim^1^ 1 Department of Anesthesiology and Pain Medicine, Seoul National University Hospital, Seoul National University College of Medicine, Seoul, Republic of Korea 2 Department of Anesthesiology and Pain Medicine, Asan Medical Center, University of Ulsan College of Medicine, Seoul, Republic of Korea
Name and contact information for the trial sponsor {5b}	The Ministry of Health & Welfare, Republic of Korea National Evidence-based healthcare collaborating agency, Seoul, Republic of Korea (82-2-2174-2700)
Role of sponsor {5c}	The trial sponsor is not involved in the study design; collection, management, analysis and interpretation of data; writing of the report; the decision to submit the report for publication, and will not have authority over any of these activities.

## Introduction

### Background and rationale {6a}

Most pediatric patients require sedation for examination and procedures [1]. There are many cases where sedation is performed without an intravenous line [2]. In such cases, oral chloral hydrate is widely used for sedation [3]. However, it may cause nausea and vomiting owing to gastrointestinal irritation. Ataxia, coma, cardiac arrhythmia, and respiratory depression may occur in cases of overdose. In addition, there is concern that the metabolites of chloral hydrate, trichloroethanol, and trichloroacetic acid have carcinogenic effects [4–6]. Furthermore, the failure rate of sedation at the first attempt is relatively high (25–35%) due to unpredictable effects, slow onset, and vomiting [4–6]. Therefore, a safe and effective sedative is required to replace oral chloral hydrate.

Recently, dexmedetomidine has been increasingly used for pediatric sedation [2, 4–36]. Dexmedetomidine is a highly selective alpha-2 agonist with sedative and analgesic effects and minimal respiratory depression. An advantage of dexmedetomidine is its high transmucosal bioavailability of 65-80% [37, 38]. Intranasal dexmedetomidine can induce sedation within 30 min in pediatric patients [2, 5, 6, 9, 11, 12, 14–22, 24, 26–28, 31, 35, 36, 38, 39] .A recent study reported that intranasal dexmedetomidine 4 μg/kg enabled magnetic resonance imaging (MRI) examination without apnea and hypoxemia in infants [27]; however, 30.7% (16/52) of the patients developed bradycardia (< 80% of the baseline heart rate) and 3.8% (2/52) developed hypotension (< 80% of the baseline mean arterial pressure). As such, intranasal dexmedetomidine alone can be associated with slow onset time, bradycardia, or hypotension.

Ketamine is an n-methyl-d-aspartate (NMDA) antagonist widely used for pediatric sedation. Ketamine preserves respiration and airway reflexes, stimulates heart function with increased blood pressure, and induces moderate bronchodilation. Intranasal ketamine has a relatively fast onset time of 3–12 min compared with intranasal dexmedetomidine [39]. Accordingly, ketamine and dexmedetomidine can complement each other upon combined administration.

As a premedication before anesthesia, the combination of intranasal dexmedetomidine and oral ketamine is more effective than each medication alone [23]. A nebulized combination of low-dose ketamine and dexmedetomidine produced more satisfactory sedation and provided a smoother induction of general anesthesia than nebulized ketamine or dexmedetomidine alone, with more rapid recovery and no significant side effect [13]. A retrospective analysis showed that the combination of intranasal dexmedetomidine (2 μg/kg) and ketamine (1 mg/kg) induced procedural sedation with a success rate of 93%, median onset time of 15 min, and median recovery time of 45 min [2]; however, the success rate was 60% for MRI sedation, and only 32.4% responded to rescue sedation with a half-dose (intranasal dexmedetomidine 2 μg/kg and ketamine 1 mg/kg).

### Objectives {7}

This study aims to determine the efficacy and safety of an intranasal combination of dexmedetomidine (2 μg/kg) and ketamine (3 mg/kg) compared to oral chloral hydrate (50 mg/kg). The primary outcome is the success rate of achieving adequate sedation levels within 15 min in pediatric patients.

### Trial design {8}

This is a prospective, parallel-arm, single-blinded, Two-center, superiority randomized controlled trial with a 1:1 allocation.

## Methods: participants, interventions and outcomes

### Study setting {9}

This two-center trial will be conducted at the Seoul National University Hospital and the Asan Medical Center. Both are tertiary hospitals located in Seoul, Republic of Korea.

### Eligibility criteria {10}

We shall enroll pediatric patients aged < 7 years undergoing sedation for examination or procedures. The exclusion criteria are as follows: American Society of Anesthesiologists (ASA) classification 4–5; allergy to dexmedetomidine, ketamine, or chloral hydrate; recent medication with alpha-2 adrenergic receptor agonists or antagonists; swallowing difficulty; excessive rhinorrhea; unstable vital signs; pneumonia; upper airway infection; and when trial members consider the enrollment inappropriate.

### Who will take informed consent? {26a}

A trial member (licensed medical doctor) will explain the study protocol and obtain written informed consent from the parents or guardians of each patient. Participants will be screened and informed consent will be obtained on the day of sedation.

### Additional consent provisions for the collection and use of participant data and biological specimens {26b}

We shall not collect biological specimens from the participants in this study. We shall store patient data in the pediatric sedation registry (http://pedisedation.com) for future analysis. One of the trial members will explain the enrollment in the pediatric sedation registry and obtain additional written informed consent from the parents or guardians of each patient.

### Interventions

#### Explanation for the choice of comparators {6b}

We shall randomly allocate patients to the intervention group (intranasal combination of dexmedetomidine 2μg/kg and ketamine 3 mg/kg, IN DEXKET) and the control group (chloral hydrate 50 mg/kg). We chose oral chloral hydrate of 50 mg/kg as the comparator because it is commonly used for pediatric sedation without an intravenous line worldwide. There is conflicting evidence regarding the efficacy and safety of chloral hydrate and alternative sedatives in pediatric patients.

#### Intervention description {11a}

Patients will follow the hospital fasting guidelines before sedation, and the type and time of the last meal will be checked (Table 1). The baseline heart rate (HR) and oxygen saturation (SpO_2_) will be measured before the induction of sedation and continuously monitored during sedation. If the patient shows irritability or cries, HR and SpO_2_ measured in a calm, relaxed state will be used as baseline. On-site trial members will administer sedatives according to the group allocation. In the intervention (IN DEXKET) group, the parents will clear the patient’s nose and hold them in their arm to keep the head supine to facilitate intranasal absorption. After administering a combination of dexmedetomidine 2 μg/kg (100 μg/mL) and ketamine 3 mg/kg (50 mg/mL) using an intranasal mucosal atomizer device (SEDACO, Doowon Meditec, Gyeonggi, Republic of Korea), the nose will be gently massaged to enhance absorption. In the control group, chloral hydrate syrup 50 mg/kg (100 mg/mL) will be administrated orally. Patient acceptance of drug administration will be assessed using a 4-point scale (1 = excellent, can be administered without difficulty; 2 = good, brief tears or grimacing, but can be administered; 3 = fair, does not cooperate but can be administered; 4 = poor, refusal of medication).

**Table 1:** Fasting guideline for pediatric sedation (Seoul National University Hospital)

Ingested material	Minimum fasting period
Clear fluid	Allowed (small amount)
Breast milk	2 h
Infant formula, nonhuman milk	4 h
Solid food	4 h

#### Criteria for discontinuing or modifying allocated interventions {11b}

If continuing the study protocol affects patient safety, the allocated intervention will be discontinued. If the parents withdraw the informed consent, or if the reliability of data is compromised, the participants will be dropped from the study.

#### Strategies to improve adherence to interventions {11c}

Not applicable; the designated intervention will be applied in a single procedural sedation.

#### Relevant concomitant care permitted or prohibited during the trial {11d}

Not applicable; no relevant concomitant care will be permitted or prohibited.

#### Provisions for post-trial care {30}

The principal investigator has clinical trial liability insurance that is in accordance with the legal requirements in Korea. This insurance provides coverage for any damage to the research participants through injury or death caused by the study. Insurance applies to damages that become apparent during the study or within 1 year after the end of the study. The principal investigator has clinical trial liability insurance following the legal requirements of the Republic of Korea. This insurance covers medical damage caused by the study to research participants through injury or death. The insurance applies to damages that occurred during the study protocol or within 2 h after the end of sedation.

#### Outcomes {12}

The primary outcome will be the success rate of sedation (Pediatric Sedation State Scale, PSSS = 1, 2, or 3) within 15 min of sedative administration [40]. We contacted the developers of the PSSS, obtained permission to translate it into Korean, and adapted it to the participating hospitals. The PSSS was educated on pediatric sedation providers including 7 pediatric anesthesiologists and 6 pediatric sedation nurses. All raters were given 30 min of PowerPoint presentation including 8 video clips showing variable sedation states.

Secondary outcomes include PSSS and vital signs during sedation, overall sedation profile, and adverse outcomes during sedation. The detailed secondary outcomes are presented in Table 2.

**Table 2:** Outcome variables

**Primary outcome**	
Success rate of sedation (PSSS = 1, 2, or 3) within 15 min from sedative administration	
**Secondary outcome**	
Onset time of sedation (PSSS = 1, 2, or 3)	
Duration of sedation, from onset time to recovery time (PSSS = 4 or 5)	
Sedation failure (PSSS = 4 or 5) after 30 min from administration	
Rescue sedation agent (or method)	
Type of procedure	
Completion of procedure	
Causes of procedure failure	
Total cost of sedation	
PSSS, heart rate, respiration rate, SpO_2_ at baseline and every 10 min (blood pressure is measured when heart rate is < 70% of baseline)	
The incidence of PSSS = 0 (abnormal physiologic parameter that require acute intervention)	
The incidence of significant apnea (> 20 s)	
The incidence of significant desaturation (SpO_2_ < 95% or < − 10% from baseline, > 10 s)	
The incidence of respiratory intervention: manual ventilation or artificial airway	
The lowest SpO_2_ value (%)	
The incidence of significant bradycardia (− 30% from baseline)	
The incidence of significant hypotension (− 30% from baseline)	
The incidence of hemodynamic intervention (fluid management, intravenous medication)	
Patients’ acceptance (1 = excellent, 2 = good, 3 = fair, 4 = poor)	
Separation anxiety (1 = easy, 2 = whimper, 3 = cry, 4 = cry and cling to parents)	
Physicians’ satisfaction (1 = excellent, 2 = good, 3 = fair, 4 = poor)	
Other side effects (e.g., nausea/vomit, allergic reaction)	

*PSSS* Pediatric Sedation State Scale [40], *SpO*_*2*_ oxygen saturation

#### Participant timeline {13}

**Fig. 1:**
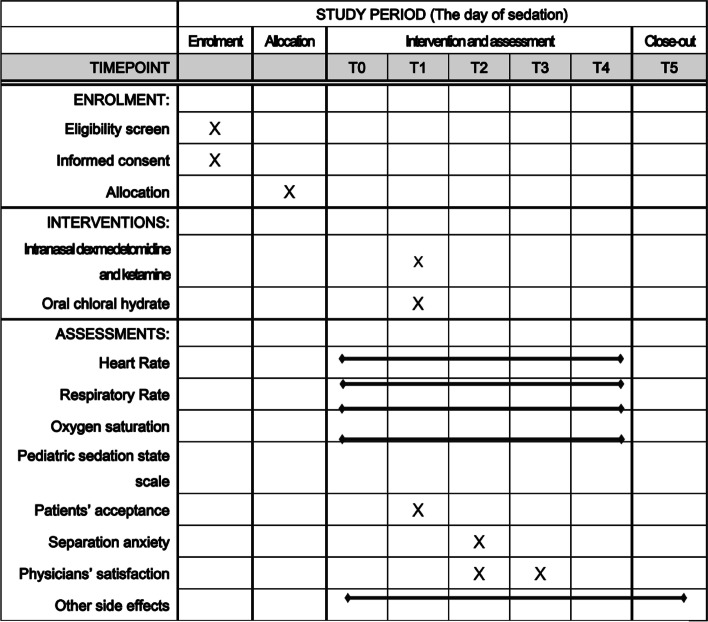
Schedule for enrollment, interventions, and assessment. T0, baseline before administration of sedative; T1, administration of sedative; T2, when the procedure starts; T3, when the procedure ends; T4, when the patients wakes up (pediatric sedation state scale = 4, or 5), T5, 2 h from T4

Participant timeline is shown in Fig. 1.

#### Sample size {14}

The primary outcome is the success rate of sedation (PSSS = 1, 2, or 3) within 15 min of sedative administration. Previous studies have reported that the success rate of sedation with oral choral hydrate (50 mg/kg) within 30 min is 65–75% [4–6]. We assumed that the success rate of oral chloral hydrate sedation within 15 min is 55%, and the success rate in the intervention group is 80%. With an alpha error of 5% and a power of 85%, 122 patients with a 1:1 allocation to the intervention and control groups will be required. Assuming a dropout rate of 10%, we planned to enroll 136 patients.

#### Recruitment {15}

On-site trial members with a medical doctor’s license (YE Jang, EY Ju) will make a list of patients scheduled to undergo procedural sedation at Seoul National University Hospital and the Asan Medical Center on the day of sedation. They will visit the candidate’s parents in the wards or outpatient clinic, check the inclusion and exclusion criteria, explain the study protocol, provide at least 20 min of pondering, and obtain written informed consent from the parents or guardians.

### Assignment of interventions: allocation

#### Sequence generation {16a}

The Medical Research Collaborating Center (MRCC) of Seoul National University Hospital offers a web-based randomization solution (https://mrcc.snuh.org/). Stratified block randomization will be performed according to age (< 1 year and 1–7 years) and location (Seoul National University Hospital and Asan Medical Center) with an allocation ratio of 1:1.

#### Concealment mechanism {16b}

A nurse who is not participating in the study will keep the randomization table on a web-based randomization site. The allocation will be concealed until the last participant’s study protocol ends.

#### Implementation {16c}

After enrollment of a participant, a nurse who is not participating in the study will log into the web-based randomization site and check the inclusion and exclusion criteria of the participant. After verifying the date of birth and the inclusion and exclusion criteria, the results of randomization will be detected and stored on the web-based randomization site. The nurse will prepare the studied drug according to the group allocation.

### Assignment of interventions: blinding

#### Who will be blinded {17a}

An independent outcome assessor, a pediatric sedation nurse not involved in group randomization or drug administration, will record the HR and SpO_2_ and assess sedation level using a 6-point PSSS. Care providers and data analysts will be blinded to the group allocation.

#### Procedure for unblinding if needed {17b}

On-site trial members who administer sedatives will not be blinded. Unblinding is allowed for the occurrence of any serious adverse event that forces the participant to drop out of the study and is determined by the trial steering committee. When the unblinding of a specific patient is decided, the on-site trial member who can access the web-based randomization table will be asked to review the table and tell the group allocation to the trial steering committee and caregivers.

### Data collection and management

#### Plans for assessment and collection of outcomes {18a}

An independent outcome assessor, a pediatric sedation nurse not involved in group randomization or drug administration, will record the HR and SpO_2_ and will assess sedation level using a 6-point PSSS (Table 2) [40]. PSSS = 4 and 5 are defined as awake states and PSSS = 1, 2, and 3 are defined as sedated states (Table 3). After administering sedatives, the HR, SpO_2_, and PSSS will be recorded every 10 min. In addition, HR, SpO_2_, and PSSS will be recorded 15 min after administration of sedative (primary outcome), at the induction of sedation (the first time of PSSS ≤ 3), at recovery from sedation (PSSS ≥ 4), and when the cardiorespiratory event occurs. The primary outcome is sedation induction (PSSS ≤ 3) within 15 min of sedative administration. If the PSSS is 0, 4, or 5 after 30 min, it is defined as a failure to induce sedation.

**Table 3:** Six-point Pediatric Sedation State Scale (PSSS) [40]

State	Behavior
5	Patient is moving (purposefully or non-purposefully) in a manner that impedes the proceduralist and requires forceful immobilization. This includes crying or shouting during the procedure, but vocalization is not required. Score is based on movement.
4	Moving during procedure (awake or sedated0 that requires gentle immobilization for positioning, may verbalize some discomfort or stress, but there is no crying or shouting that expresses stress or objection.
3	Expression of pain or anxiety on face (may verbalize discomfort), but not moving or impending completion of the procedure. May require help positioning (as with a lumbar puncture) but does not require restraint to stop movement during the procedure.
2	Quiet (asleep or awake), not moving during procedure, and no frown (or brow furrow) indicating pain or anxiety. No verbalization of any complaint.
1	Deeply asleep with normal vital signs, but requiring airway intervention and/or assistance (e.g., central or obstructive apnea, etc.)
0	Sedation associated with abnormal physiologic parameters that require acute intervention (i.e., oxygen saturation < 90%, blood pressure 30% lower than baseline, bradycardia receiving therapy)

If the sedation level is inadequate (PSSS = 4 or 5) for procedures or examinations, the attending medical staff will decide on the method of rescue sedation. As pain during blood pressure measurement can wake up the patient, blood pressure will be measured when the HR is less than 70% of the baseline value. If the patient shows significant bradycardia or hypotension (− 30% from baseline), the trial member will offer hemodynamic intervention, such as fluid management and intravenous medication. If the patient shows apnea for ≥ 20 s or a significant decrease in pulse oxygen saturation (SpO2 < 95% or < 90% from baseline value ≥ 10 s) occurs, the trial member will secure the airway and apply appropriate stimulation for recovery.

Before the examination or procedure, separation anxiety will be evaluated using a 4-points scale (1 = easy, easily separated from parent; 2 = whimper, whimpering but separated; 3 = crying and reluctant to separate but not clinging; and 4 = crying and cling to parents; crying and clinging to parent). Successful completion of the procedure will be recorded, and if stopped, the reason will be recorded. Physician satisfaction will be evaluated using 4 scales. (1 = excellent, fearless, and cooperative with the test; 2 = good, showing some fear but reassuring quickly when appeased; 3 = fair, showing fear and not being appeased; 4 = poor, showing fear or crying or resistance to tests or procedures). When the examination or procedure ends, the patient will be transferred to the recovery room or ward bed. The time when PSSS = 4 will be recorded to check the duration of sedation.

#### Plans to promote participant retention and complete follow-up {18b}

The study timeline will be completed on the day of sedation. There is no follow-up.

#### Data management {19}

**Fig. 2:**
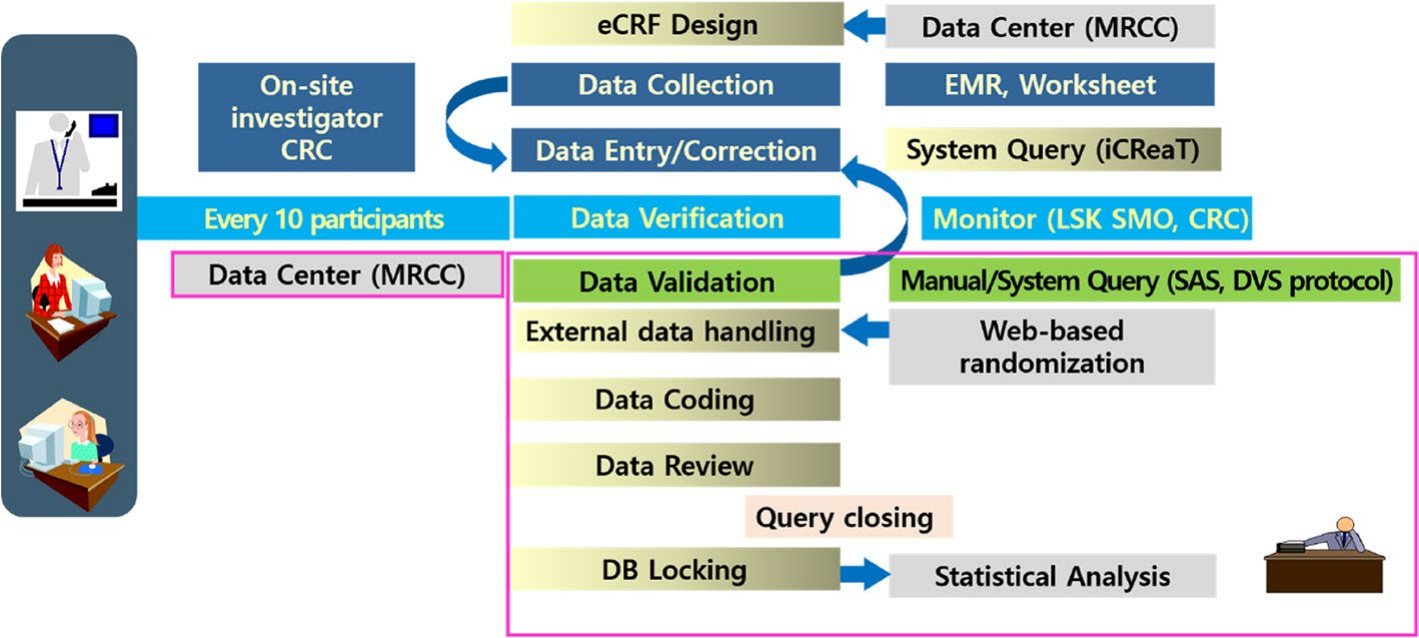
Data management flow. CRC, clinical research coordinator; MRCC, medical research collaborating center; EMR, electronic medical record; LSK SMO, LSK site management organization; DVS, data validation specification; DB, database

On-site investigators (YE Jang at Seoul National University Hospital and EY Ju at Asan Medical Center) are responsible for recruiting patients, conducting the study, and recording data on the worksheet (paper-based case report form). The records will be submitted to the Internet-based Clinical Research and Trial Management System (http://icreat.nih.go.kr), an electronic case report form (eCRF), by the National Institute of Health, Korea. The patients will be identified using a unique numeric code, and all patient data will be anonymized to ensure patient confidentiality. All eCRF data management will be conducted by the MRCC, Seoul, Korea. Data monitoring (verification) of paper-based case report form and eCRF will be conducted when every 10 patients are enrolled, by the Clinical Research Coordinator and the LSK Site Management Organization, a Contract Research Organization in Seoul, Korea. Data validation will be performed using automatic query by SAS edit checks and manual query, data clarification, and query resolution by MRCC. External data handling will be performed by checking the consistency of the web-based randomization, protocol deviation log, and eCRF data. The latest version of the data management plan is 1.1 (June 7, 2021), and that of the data validation specification is 1.1 (April 8, 2022) (Fig. 2).

#### Confidentiality {27}

Patients will only be referred to by the number of participants. Written informed consent and paper-based case report forms will be stored separately. Data will be stored within a double-locker until the expiration of the storage period and will only be accessible to trial members. After a storage period of 3 years, we shall destroy the data.

#### Plans for collection, laboratory evaluation, and storage of biological specimens for genetic or molecular analyses for this trial/future use {33}

Not applicable. No biological specimen will be collected for laboratory testing during the trial.

### Statistical methods

#### Statistical methods for primary and secondary outcomes {20a}

All data will be expressed as mean ± standard deviation or median (interquartile range [IQR]) unless otherwise specified. We shall test distribution using the Shapiro-Wilk normality test and evaluate the baseline characteristics of the study population using the independent *t-*test and Mann-Whitney *U* test. We shall analyze outcomes according to the intention-to-treat principle. The primary outcome (the success rate of sedation (PSSS = 1, 2, or 3) within 15 min of sedative administration) will be evaluated using the *χ*^*2*^ test, while secondary outcomes will be evaluated using the *χ*^*2*^ test, independent *t*-test, and Mann-Whitney *U* test. Statistical analyses will be performed using IBM® SPSS® Statistics 23 (IBM Corp., Armonk, NY, USA) and R software (version 3.4.4; R Foundation for Statistical Computing, Austria). Statistical significance shall be set as a two-sided *p* < 0.05. To evaluate safety, we shall monitor and collect HR, SpO_2_, and RR throughout the study as secondary outcomes. For repeated-measured data, analysis using a mixed model shall be performed to compare the two groups over time. It is expected that there will be a few missing values for the primary endpoint, and missing values will not be substituted for primary and secondary evaluations.

#### Interim analyses {21b}

No interim analysis will be conducted during this trial.

#### Methods for additional analyses (e.g., subgroup analyses) {20b}

Not applicable; no additional analysis is intended for this study.

#### Methods of analysis to handle protocol non-adherence and any statistical methods to handle missing data {20c}

Not applicable; the written informed consent and all data will be obtained on the day of sedation. There will be no non-adherence issue in this study.

#### Plans to give access to the full protocol, participant-level data, and statistical code {31c}

Sharing the data can be considered by the corresponding author on reasonable request.

### Oversight and monitoring

#### Composition of the coordinating center and trial steering committee {5d}

The coordinating center is the Department of Anesthesiology and Pain Medicine of Seoul National University Hospital, Seoul, Korea. The trial steering committee consists of the principal investigator (JT Kim) and other investigators (YE Jang and EY Ju) who are responsible for recruiting patients, conducting the study, and data entry.

#### Composition of the data monitoring committee, its role and reporting structure {21a}

Data monitoring (verification) will be conducted by the clinical research coordinator and the LSK site management organization, a contract research organization in Seoul, Korea. Data validation and external data handling (web-based randomization) will be conducted by statisticians and data managers at the MRCC in Seoul, Korea. The safety of the participants and the appropriateness of the data will be monitored and reported every three months and under the supervision of the principal investigator.

#### Adverse event reporting and harms {22}

The principal investigators (JT Kim at Seoul National University Hospital, and EY Ju at Asan Medical Center) will report any serious adverse events to each institutional review board within 15 days of the notice. Mild adverse events will be reported every 3 months. When reporting adverse events or harm, we shall equally report causalities, time of occurrence, severity, seriousness, provided management, and the relationship to the present clinical trial.

#### Frequency and plans for auditing trial conduct {23}

Self-auditing will be performed by the site management organization every three months. The trial may be audited at any time by the Ministry of Food and Drug Safety of Korea or the institution’s quality assurance team.

#### Plans for communicating important protocol amendments to relevant parties (e.g., trial participants and ethical committees) {25}

Any amendment to the protocol will be reviewed by the Institutional Review Board of Seoul National University Hospital and the Asan Medical Center Institutional Review Board. After approval, the changes will be reported and reviewed by the Ministry of Food and Drug Safety of Korea. These changes will be reflected in the public registry site (https://clinicaltrials.gov/ct2/show/NCT04820205).

#### Dissemination plans {31a}

The results will be disseminated via publication in a peer-reviewed journal, available at the registry site (https://clinicaltrials.gov/ct2/show/NCT04820205) and presented at scientific meetings.

## Discussion

### Determination of intranasal dexmedetomidine and ketamine dose

The intranasal combination of dexmedetomidine and ketamine will be used to (1) shorten the onset and recovery time of sedation, (2) avoid bradycardia or hypotension, and (3) provide analgesia for mild pain or discomfort during procedures.

Dexmedetomidine is a selective alpha-2 adrenergic agonist with sedative, anxiolytic, analgesic effects, and minimal respiratory depression. It has a relatively high transmucosal bioavailability of 65–80% [15]. Therefore, dexmedetomidine has been successfully used for pediatric sedation. Clinically, intranasal dexmedetomidine at 1–3 μg/kg has an onset of 20–30 min, and the duration of action is 30–60 min [26]. Therefore, the onset time must be shortened to ensure efficient sedation. The plasma concentration of intravenous dexmedetomidine in sedated intensive care unit patients is 400–800 pg/mL [36]. Peak plasma concentration is 199 pg/mL 46 min after intranasal dexmedetomidine 1 μg/kg and increases to 355 pg/mL 47 min after intranasal dexmedetomidine 2 μg/kg. Therefore, high dose intranasal dexmedetomidine shows a quicker onset and greater analgesic effect; however, the duration of action is prolonged and the possibility of side effects, such as bradycardia or hypotension, increases. Intranasal dexmedetomidine 4 μg/kg showed successful sedation for MRI in 96.2% (49/52) of infants without motion artifacts, apnea, or desaturation. However, bradycardia (< 80% of the baseline heart rate) occurred in 30.7% (16/52), and hypotension (< 80% of the baseline mean arterial pressure) occurred in 3.8% (2/52) [27].

Ketamine has sedative, anxiolytic, and analgesic effects while preserving the airway reflex. It shows cardiovascular stability with anticholinergic properties, such as tachycardia and bronchodilation. According to a recently published systematic review article (25 studies comprising 161 patients) [39], intranasal ketamine can be administered at various doses 0.5–10 mg/kg for sedation, painful procedures, radiology tests, and premedication for anesthesia. Intranasal ketamine up to 10 mg/kg offers effective sedation and analgesia without serious side effects. According to this review, intranasal ketamine 3–9 mg/kg-induced sedation had an onset of 3.6–11.6 min and lasted for 7–69 min.

We determined the dose of intranasal dexmedetomidine (2 μg/kg) and intranasal ketamine (3 mg/kg), considering the different onsets and durations of the two drugs for safe synergetic sedation with rapid onset and sustainability. In addition, we expect a hemodynamic balance between dexmedetomidine-induced bradycardia and ketamine-induced tachycardia.

### Adopting the pediatric sedation state scale

The assessment tool for sedation is one of the key features of the present study. Before the present study, one of the participating hospitals used the Ramsay Sedation Scale to assess pediatric patients [41]; however, the scale was initially developed, evaluated, and used for adult patients in the intensive care unit. Therefore, we sought to determine an optimal scale for pediatric procedural sedation in the present study.

In pediatric patients, previously used sedation scales focused on the depth of sedation during the procedure. However, successful procedural sedation requires control of movements and blunting of noise and pain, without respiratory depression or hemodynamic instability. The PSSS was developed and validated to assess the quality of procedural sedation, including the control of pain, anxiety, movement, and adverse events [40]. The authors enrolled 99 children aged 1–7 years who underwent laceration repair with intranasal midazolam. The PSSS state (0–5) describes the movements (need for immobilization or positioning), expression of anxiety and pain, and the need for intervention (respiration, blood pressure, or heart rate). The PSSS showed excellent interrater and intrarater reliabilities with no more than 10 min of teaching. We contacted the developers of the PSSS, obtained permission to translate it into Korean, and adapted it to the participating hospitals. (Table 4.) However, the Korean version of PSSS is not validated yet and needs further study for wide use in Korea.

**Table 4:** Six-point Pediatric Sedation State Scale (PSSS) in Korean

State	Behavior
5	환자가 의도적 또는 비의도적인 움직임을 보이고 시술을 방해해서 강제적인 고정이 필요함. 시술 도중 울거나 소리치는 경우가 포함되지만, 발성이 반드시 있어야 하는 것은 아님. 점수는 움직임을 기반으로 판단함.
4	시술 도중 움직임이 있어 자세를 유지하기 위해 가벼운 고정이 필요함. 불편함이나 스트레스를 말로 표현할 수 있으나, 울거나 소리치지는 않음. 깨어 있거나 진정 상태 모두 가능.
3	얼굴에 통증 또는 불안을 표시하고 불편함을 말할 수 있으나, 움직이거나 시술을 막으려고 하지 않음. 자세 유지를 위해 도움이 필요할 수는 있으나(예. 척추 천자 등), 시술 도중 움직임을 멈추기 위한 제한이 필요하지는 않음.
2	시술 중 조용한 상태를 유지하고 움직이지 않으며, 통증이나 불안을 나타내는 찡그림이 없음. 어떠한 불만을 이야기하지 않음. 깨어 있거나 수면 중인 상태 모두 가능.
1	깊이 잠든 상태로 활력징후는 정상이나 기도 조작 또는 호흡 보조가 필요함. (예. 중추성 또는 폐쇄성 무호흡증)
0	즉시 처치가 필요한 비정상적인 활력 징후를 보이는 상태의 진정 (예. 산소포화도 90% 미만, 기저치보다 혈압이 30% 이상 감소, 치료가 필요한 서맥)

## Limitation

Our study has some limitations. First, the doses of intranasal dexmedetomidine (2 μg/kg) and ketamine (3 mg/kg) were determined for procedures that require minimal analgesia and a short duration (< 1 h). Therefore, it is not suitable for procedures with moderate pain or prolonged time (e.g., insertion of a central venous catheter or MRI of the brain and whole spine). Second, placebo administration (intranasal saline in the chloral hydrate group and oral saline in the intranasal group) is not used in this study, and patients and parents would not be blinded. Because oral and intranasal administrations are stressful to the participants, we decided not to use placebo administration. Instead, we recorded patients’ acceptance (1 = excellent, 2 = good, 3 = fair, 4 = poor) during sedative administration to measure compliance. Third, the authors could not find a validated tool to assess the separation anxiety and physician satisfaction scale in Korean for pediatric sedation, and the questionnaires used in the present study are not validated.

In summary, we expect that this study will provide evidence for safer and more efficient procedural sedation in pediatric patients.

## Trial status

The final protocol version is 1.3 (August 11, 2021), and the first patient was recruited on October 29, 2021. Recruitment is expected to be complete by December 2024. For the final analysis, the database will be cleaned and checked for completeness, before being locked and statistical analysis carried out.

Final protocol version: v1.3 (date: August 11, 2021)

Recruitment of the first patient: October 29, 2021

Number of patients recruited: 62 (date: September 6, 2022)

Expected date for completion of recruitment: December 31, 2024

## Data Availability

The datasets generated by the current study protocol are available from the corresponding author upon reasonable request.

## Abbreviations

MRI: Magnetic resonance imaging
NMDA: n-methyl-d-aspartate
ASA: American society of anesthesiologists
IN DEXKET: Intranasal combination of dexmedetomidine and ketamine
HR: Heart rate
SpO2: Oxygen saturation
PSSS: Pediatric Sedation State Scale
MRCC: Medical Research Collaborating Center
eCRF: Electronic case report form
